# A pilot cost-analysis study comparing AI-based EyeArt® and ophthalmologist assessment of diabetic retinopathy in minority women in Oslo, Norway

**DOI:** 10.1186/s40942-024-00547-3

**Published:** 2024-05-23

**Authors:** Mia Karabeg, Goran Petrovski, Silvia NW Hertzberg, Maja Gran Erke, Dag Sigurd Fosmark, Greg Russell, Morten C. Moe, Vallo Volke, Vidas Raudonis, Rasa Verkauskiene, Jelizaveta Sokolovska, Inga-Britt Kjellevold Haugen, Beata Eva Petrovski

**Affiliations:** 1 Center for Eye Research and Innovative Diagnostics, Department of Ophthalmology, Institute for Clinical Medicine, University of Oslo, Kirkeveien 166, 0450 Oslo, Norway; 2 Department of Ophthalmology, Oslo University Hospital, Kirkeveien 166, 0450 Oslo, Norway; 3 Department of Ophthalmology, University of Split School of Medicine and University Hospital Centre, 21000 Split, Croatia; 4 UKLONetwork, University St. Kliment Ohridski-Bitola, 7000 Bitola, Macedonia; 5 Clinical Development, Eyenuk Inc, Woodland Hills, CA USA; 6 Norwegian Association of the Blind and Partially Sighted, Oslo, Norway; 7 Faculty of Medicine, University of Latvia, Jelgavas street 3, LV1004 Riga, Latvia; 8 Faculty of Medicine, Tartu University, 50411 Tartu, Estonia; 9 Automation Department, Kaunas University of Technology, 51368 Kaunas, Lithuania; 10 Institute of Endocrinology, Lithuanian University of Health Sciences, 50161 Kaunas, Lithuania

**Keywords:** Screening, Diabetic retinopathy, Minority women, Norway, Manual grading, Automated grading, Artificial intelligence, Cost-analysis

## Abstract

**Background:**

Diabetic retinopathy (DR) is the leading cause of adult blindness in the working age population worldwide, which can be prevented by early detection. Regular eye examinations are recommended and crucial for detecting sight-threatening DR. Use of artificial intelligence (AI) to lessen the burden on the healthcare system is needed.

**Purpose:**

To perform a pilot cost-analysis study for detecting DR in a cohort of minority women with DM in Oslo, Norway, that have the highest prevalence of diabetes mellitus (DM) in the country, using both manual (ophthalmologist) and autonomous (AI) grading. This is the first study in Norway, as far as we know, that uses AI in DR- grading of retinal images.

**Methods:**

On Minority Women’s Day, November 1, 2017, in Oslo, Norway, 33 patients (66 eyes) over 18 years of age diagnosed with DM (T1D and T2D) were screened. The Eidon - True Color Confocal Scanner (CenterVue, United States) was used for retinal imaging and graded for DR after screening had been completed, by an ophthalmologist and automatically, using EyeArt Automated DR Detection System, version 2.1.0 (EyeArt, EyeNuk, CA, USA). The gradings were based on the International Clinical Diabetic Retinopathy (ICDR) severity scale [1] detecting the presence or absence of referable DR. Cost-minimization analyses were performed for both grading methods.

**Results:**

33 women (64 eyes) were eligible for the analysis. A very good inter-rater agreement was found: 0.98 (*P* < 0.01), between the human and AI-based EyeArt grading system for detecting DR. The prevalence of DR was 18.6% (95% CI: 11.4–25.8%), and the sensitivity and specificity were 100% (95% CI: 100–100% and 95% CI: 100–100%), respectively. The cost difference for AI screening compared to human screening was $143 lower per patient (cost-saving) in favour of AI.

**Conclusion:**

Our results indicate that The EyeArt AI system is both a reliable, cost-saving, and useful tool for DR grading in clinical practice.

## Introduction

Diabetes mellitus (DM) is a major medical and societal challenge due to its rapid increase in global prevalence and devastating late complications. A dramatic rise in the number of new cases is expected due to an overweight epidemic and an ageing population during the next decades [2, 3]. In Norway, the estimated number of people with DM is between 316 000 to 345 000, of which, 60 000 are undiagnosed DM [4].

Diabetic retinopathy (DR) is one of the most common microvascular complications of DM [5]. It is the leading cause of secondary blindness and reduced vision in working-aged people (25 to 75 years) [5, 6]. In 2020, a study across 59 countries, found that 6.2% of people with diabetes had sight- threatening diabetic retinopathy (STR), and 4.1% had diabetic macula edema (DME), affecting totally 47.4 million individuals [5]. This number is expected to rise to 73.4 million by 2045 [7]. In Norway, the prevalence of STR was 38% in type 1 DM (T1D) and 1.5% in T2D during 2012 [8]. Regional and global programs which help lessen the burden brought on by DM are of high interest to patients, healthcare professionals and decision-makers. Such programs need systematic evaluation for their impact on health outcomes, cost-effectiveness, and health equity [9].

Systematic DR screening is proven to be cost-effective in reducing blindness and visual impairment for patients with DM by optimizing time of treatment that may halt disease progression [10]. In the Oslo region, a screening program for DR has recently been started [11] Artificial intelligence (AI) has been proven to have both high specificity and sensitivity, as well as to be cost-effective [12–15].

It is well known that some immigrant groups are more susceptible of developing T2D than native Europeans in countries they immigrate to [16, 17]. Immigrants from Asia and Africa, especially those from Pakistan and Sri Lanka [18] are at higher risk for T2D in comparison to immigrants from other parts of Europe or North America, as well as developing it at a younger age [16, 19]. Especially, the minority women population is known to have the highest prevalence of DM in Norway [18]. Ethnic minorities in Norway comprise groups of people with ancient connections to Norway (people of Finish descent (Kvens), Forest Finns, Taters, Jews) [20], and diverse immigrant groups, among others Bosnia and Herzegovina, Pakistan, Somalia and Türkiye [21]. According to the Central Statistics Bureau-Norway, there are 877 227 immigrants in Norway in 2023 (7.1% more than the year before). Together with Norwegian-born to immigrant parents, this group made up to 19.9% of the population of Norway [22]. Non-European immigrants mainly come to Norway from Asia (31%), Africa (13%) North & South America and Oceania (5%).

Here, we report the findings from a cross-sectional pilot study of an internal quality register in Oslo, Norway, which utilizes AI in the screening of retinal images for DR. The aim of this study was to perform a cost-analysis of the detection of DR in a cohort of minority women with DM in Oslo, Norway, using both manual/ophthalmologist- (gold standard) and AI-grading of DR. As Norway has not yet established an AI screening financing system, we used a new establishment in the USA as a comparator to human screening in Norway [23].

## Patients and methods

### Study setup

This pilot study was conducted in accordance with the Declaration of Helsinki and approved by the Data Protection Officer (DPO) at Oslo University Hospital (OUH), No. 20/11,953. All participants took part in the study on a voluntary basis and anonymously. In cases where STR was detected, the patients were referred for treatment at the Department of Ophthalmology, Oslo University Hospital, Oslo, Norway.

Consecutive patients with self-reported DM (T1D and T2D) were screened on the Minority Women Day on November 1, 2017, in Oslo, Norway. Thirty-three [33] patients (66 eyes)who expressed interest in having their photos taken, were examined and graded promptly over a period of 2 h.

The inclusion criteria of the study were: all participants who attended the screening day; 18 years of age and older; diagnosis of diabetes; willingness to undergo examination. The enrolment criteria did not require knowledge of the patient’s ophthalmic history, including DR diagnosis. The exclusion criteria were: strabismus; opaque cornea; continuous vision loss in one or both eyes; inability to communicate and understand the instructions. Images were collected anonymously, so no personal data were collected.Fundus images, 2per eye, were obtained by the same qualified nurse using non-mydriatic fundus camera Eidon - True Color Confocal Scanner (CenterVue, United States) with a field size of a single photo of 60°. Images were graded for DR manually (by two experienced retina specialists) and autonomously using AI-based software (EyeArt, Eyenuk, Inc, CA, USA). The EyeArt AI system has CE marking as a class IIb medical device in the European Union under the EU’s Medical Devices Regulation 2017/745 (“MDR”) for the detection of DR, AMD, and glaucomatous optic nerve damage. It has been validated for use with color fundus photographs that provide retinal coverage equivalent to the combined coverage of field 1 (optic nerve head-centered) and field 2 (macula-centered) color fundus photographs, with a 45-degree field of view. For this particular type of camera, the eye and field (either macula or optic nerve head (ONH) center) were automatically detected by the software. Both the software quality control model and the operator confirmed the image quality.

A short explanation of the results was provided to the patients.

### Grading of diabetic retinopathy

During image evaluation, both the graders and EyeArt classified the signs and the stages of DR and maculopathy and graded the images in alignment based on the International Clinical Diabetic Retinopathy (ICDR) severity scale [24]. The scale consists of: No DR, Mild Non-proliferative DR (NPDR), Moderate NPDR, Severe NPDR, Proliferative DR (PDR) and DME. Since EyeArt system is programmed to detect referable DR (grade Moderate NPDR and above), grading by ophthalmologist was performed likewise into no DR (negative) and DR (positive), as well as the DR grades (mild, moderate, severe, proliferative) and presence or absence of diabetic maculopathy the presence of any exudates within one disc diameter from the center of the fovea.

### Statistical analysis

The weighted Kappa and Cohen’s kappa was used to test strength of agreement between the two gradings (ophthalmologist vs. AI-based grading of DR) in case of ordinal variables (grades) with 4 categories (0: no diabetic retinopathy, 1: mild; 2: moderate; 3: severe) and in case of two categories (0- No; 1- Yes). Gradings with perfect agreement would both assign the same category/grade to each fundus picture. The inter-grader agreement results were assessed using the Landis and Koch approach, where: 0.20 = poor; 0.21–0.40 = fair; 0.41–0.60 = moderate; 0.61–0.80 = good; and 0.81–1.00 = very good agreement [25]. The weighted-Kappa statistic is generally close to Spearman correlation coefficients (the Spearman’s correlation coefficient (rho) could compare the ordinal variables with 4 categories/grades ranges from 0 to 4; a Spearman`s correlation of 1 indicates a perfect, positive relationship).

### Costs and sensitivity analysis

Consistent with the National Guidelines, an extended healthcare perspective was used to calculate the total patient cost for the procedure performed in a cost-minimization analysis. This included direct- and some indirect-healthcare costs (two-way transportation and time spent to travel and receive needed care by the patient).

To estimate the direct costs for patients under manual screening, we used a diagnosis-related group (DRG) weights for the fundus photography code, which includes screening ($164). The AI screening costs were compared to those used in the USA as established in 2021 under the government health programs ($33). The autonomous diabetic retinal examination was coded as 92,229, and exact reimbursement for AI was geography-dependent, as well as other factors [23, 26]. We therefore used various ranges of values to calculate the probable total cost per patient for using AI compared to the manual/ophthalmologist screening. Indirect healthcare costs were obtained from the Norwegian Medicine Agency (NOMA 2022) database. Transport costs per journey ($73) and patient time cost per hour ($24) were averagely reported for use, including evaluation studies.

The total cost per patient was calculated by adding the estimated DRG cost values (manual/ophthalmologist screening or AI) to the transport costs and patients’ time costs. In both screening strategies, one visit was assumed, therefore a round trip cost was applied to both by multiplying the cost by 2. We also assumed the time for travel and for receiving the needed care for manual/ophthalmologist- and AI- screening being 1.5 h and 1 h, respectively. The patient time cost was multiplied with the appropriate time for each strategy. In both strategies, we assumed that the cost of the doctor or nurse doing the grading of being the same, so we excluded these costs from the calculations. AI costs were converted to U.S. dollars as per November 30, 2022, exchange rate (i.e. 9.89 Norwegian Kroner (NOK) equalling to 1 U.S. dollar).

We further estimated the total costs per patient on various ranges of values for different parameters to determine the robustness of our analysis. First, we estimated the costs viable for AI screening in a one-way deterministic analysis to establish inclusion of other factors or geographic considerations. For instance, AI cost varied from $33 to $64 while fundus photography performed within an eye clinic was reimbursed at $113, so we evaluated all these costs under the sensitivity analysis, and also checked the potential of AI reimbursement with same DRG weight used for ophthalmologist. In a two-way sensitivity analysis, we considered a range of patient time for the manual screening and AI costs. Furthermore, we estimated, in a two-way sensitivity analysis, a range of values for AI patient time and AI screening costs.

## Results

**Fig. 1:**
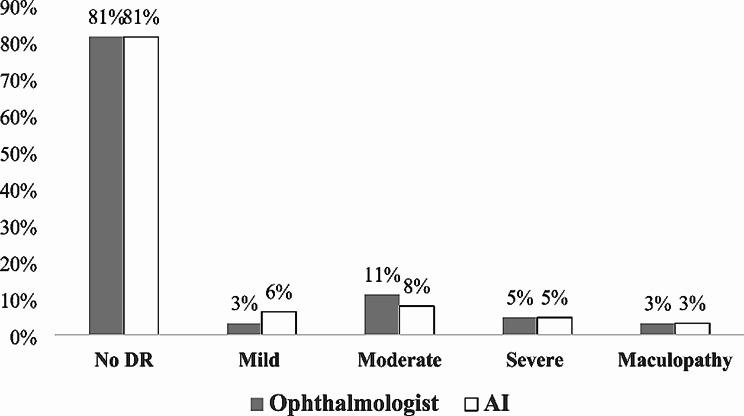
Grading of diabetic retinopathy and maculopathy in the studied population using manual/ophthalmologist- and AI-based EyeArt methods

The results of the grading for DR in the studied population by a manual/ophthalmologist- and the AI-based/EyeArt software- grading is shown in Fig. 1. Two of the 33 subjects included in the pilot study had ungradable images due to poor quality in one eye, which was accurately detected by both, manual grader and EyeArt. The distribution of the DR grades for the manual/ophthalmologist and AI-based/EyeArt was the following: 81% and 81% (No DR), 3% and 6% (Mild DR), 11% and 8% (Moderate DR), 5% and 5% (Severe DR); the presence of diabetic maculopathy was: 3% and 3%, respectively. Overall, the values did not differ significantly.

According to the results of the Weighted-kappa and, a very good inter-rater agreement was found: 0.95-0.9984 (*P* < 0.001), between the human and AI-based EyeArt grading system. The prevalence of DR and maculopathy were 18.8% (95% CI:9.1–28.3%) and 3.1% (95% CI: 0.4–10.8%), respectively; the sensitivity was 100% (95% CI: 73.5–100% and 95% CI: 15.8–100%), respectively; the specificity was 100% (95% CI: 93.1–100% and 95% CI: 94.2–100%), respectively; the accuracy was 100% (94.4–100%) and 100% (94.4–100%), respectively (Table 1).

**Table 1:** Inter-rater reliability, sensitivity/specificity and accuracy of the AI-based EyeArt grading system for diabetic retinopathy compared to manual/ophthalmologist grading

	Diabetic Retinopathy *N* = 66 eyes	Maculopathy *N* = 66 eyes
Ophthalmologist vs. AI (Weighted Kappa and Spearman’s rho)	0.95; 1.00 (*P* < 0.001)	1.00; 1.00 (*P* < 0.001)
Prevalence of Diabetic Retinopathy	18.8% (95% CI: 9.1–28.3%)	3.1% (95% CI: 0.4–10.8%)
Sensitivity	100% (95% CI: 73.5–100%)	100% (95% CI: 15.8–100%)
Specificity	100% (95% CI: 93.1–100%)	100% (95% CI: 94.2–100%)
Accuracy	100% (94.4–100%)	100% (94.4–100%)

K: weighted Cohens Kappa; CI: confidence interval; P: level of significance

The manual screening costs per patient were higher ($164) than AI screening ($33), hence human/manual screening total patient costs were similarly higher ($273) than those for AI screening ($131) at baseline. Consequently, the cost difference per patient for AI screening compared to manual screening was $143 lower (e.g. cost-saving). Considering, the estimated costs of DM patients in this study, in an extended healthcare perspective, the screening total costs for any given period would be $9009 for human screening and $4323 for AI screening. If half of the DM patients are screened by either of the strategies, the total costs will aggregate to a sum of $6666 per given screening session.

### Cost sensitivity analysis

**Fig. 2:**
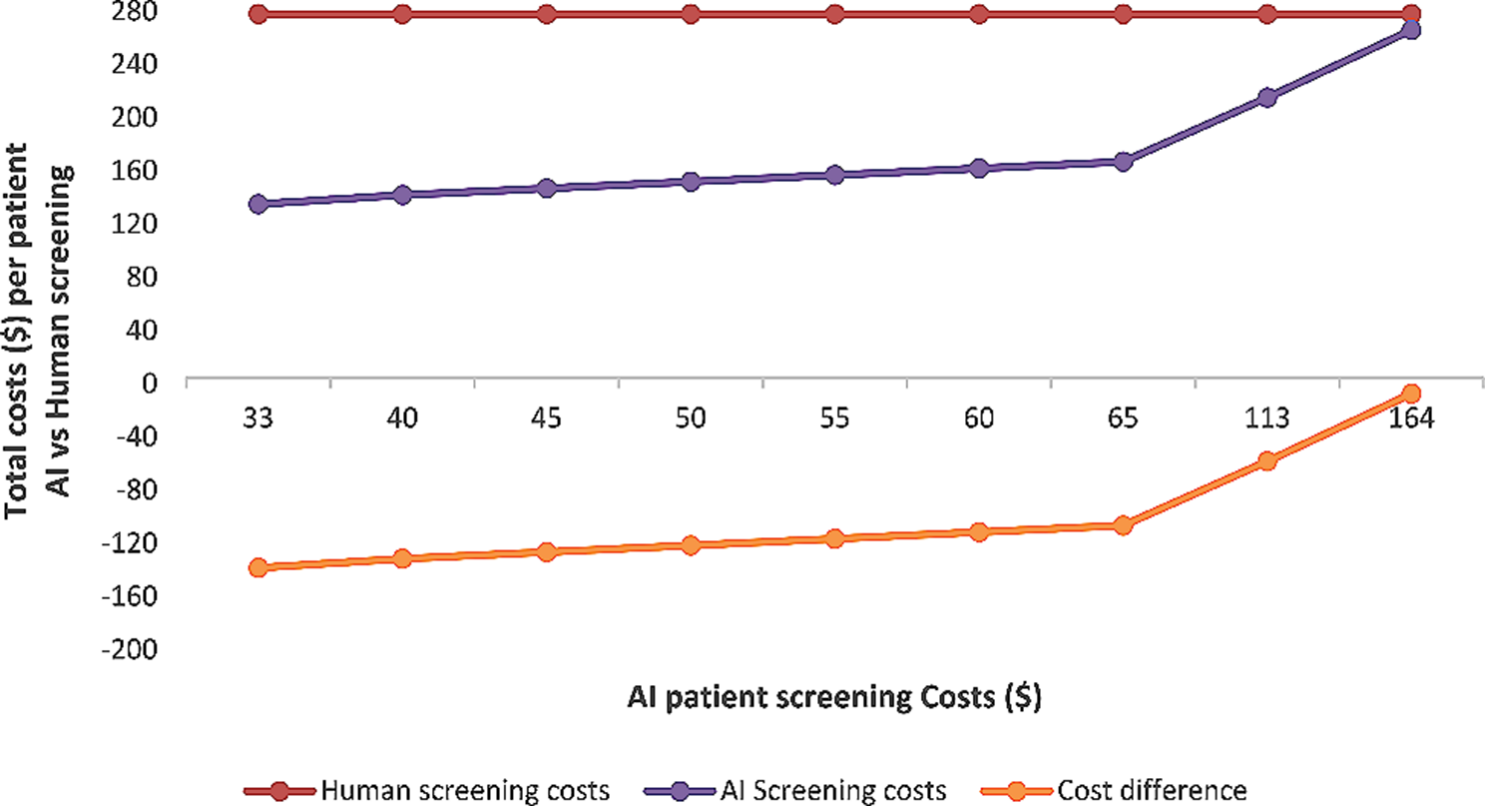
One-way sensitivity analysis of varying AI patient screening costs for the total costs and total cost difference for AI compared to human screening

The impact of the various costs on the total cost difference from an extended healthcare perspective showed that the cost saving diminished at almost equal values as manual screening costs at baseline or higher values (Fig. 2).

**Fig. 3:**
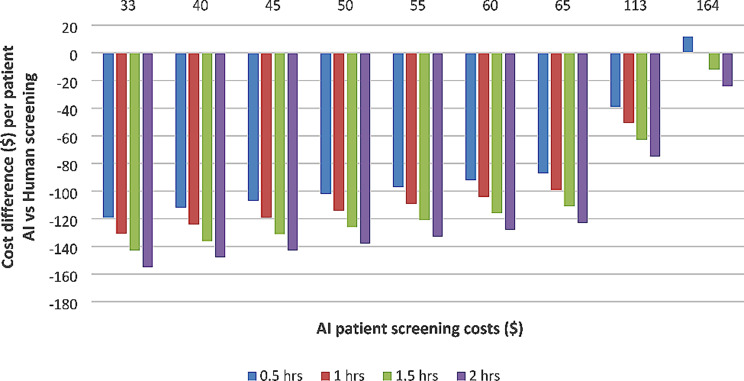
Two-way sensitivity analysis for cost difference comparing varying patient time taken from half an hour to maximum of two hours which includes travel time and time spent at the screening facility for human screening vs. AI costs

The findings remained unchanged when different times used by patients for manual screening was compared to AI costs. Thirty minutes to one hour used per patient appeared to remain cost-saving at all estimated values until the AI screening costs equaled the manual screening costs (Fig. 3). The patient time cost was multiplied with the appropriate time for each screening strategy.

**Fig. 4:**
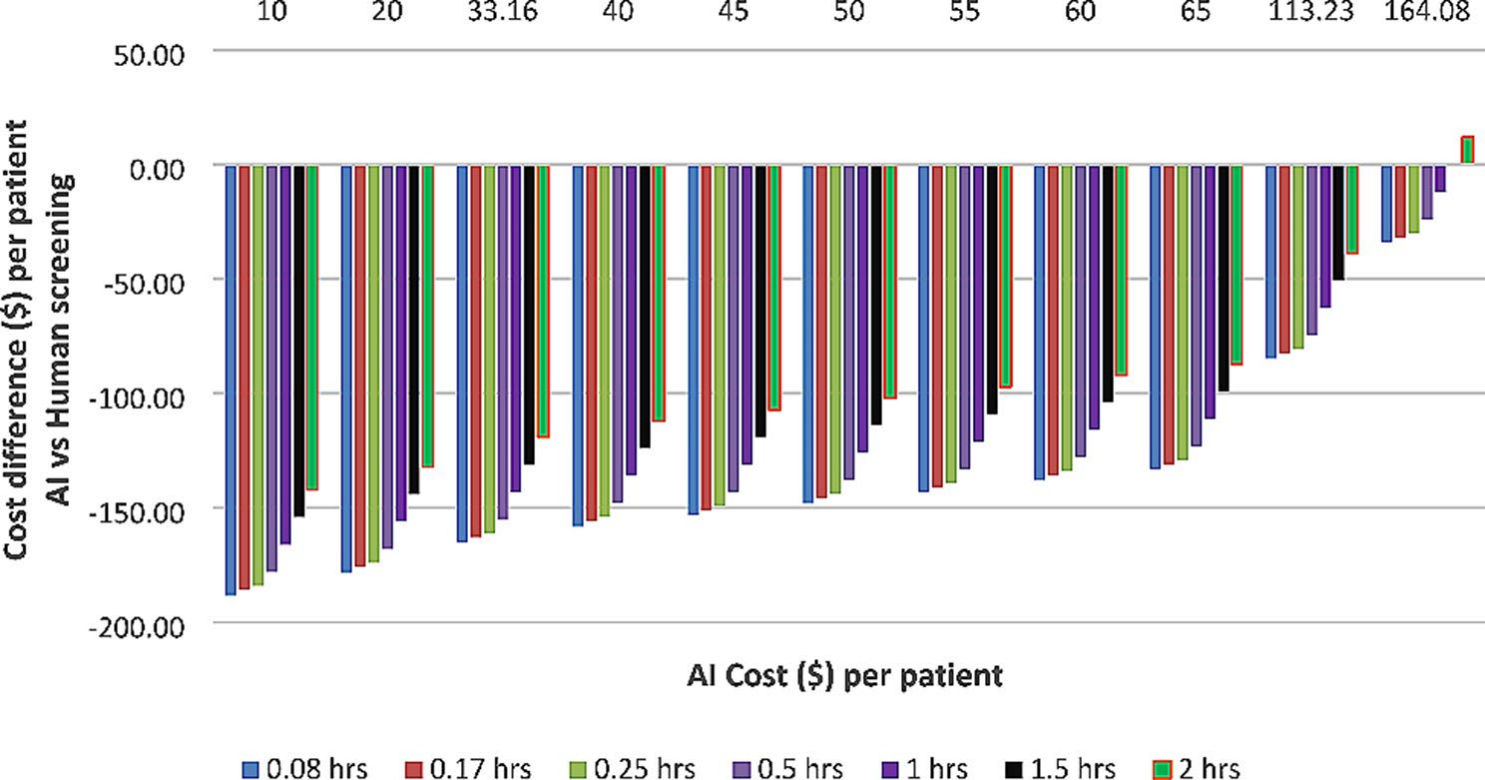
Two-way sensitivity analysis for cost difference per patient comparing varying patient time taken from five minutes to maximum of two hours which includes travel time and time spent at the screening facility for AI screening vs. AI costs

Assessing the AI screening costs and time taken by patients at the intervention found that the cost saving benefit remained despite the costs and patient time up until 2 h used for AI-based screening, and the costs were similar to manual screening (Fig. 4).

## Discussion

DR is a devastating complication of DM, which affects the working-age population, as well as the elderly, decreasing their ability to work and quality of life [27, 28]. Nevertheless, in many parts of Norway, there is no clear communication between the healthcare professionals involved in the care of patients with DM. Furthermore, the interval between eye examinations is at the ophthalmologist’s discretion. The DIABØye study investigated and interviewed about 300 Norwegian DM patients recruited from GPs, and it was estimated that only 62% had an eye examination according to the guidelines, while 26% of patients have never had an eye examination [8, 29]. In primary health care, there is a clear lack of information whether an eye examination has been performed and the result thereof [29]. Moreover, only 50.8% of T1D and 32.5% of T2Dpatients in the Norwegian diabetes registry (NOKLUS) report of regular eye examinations [30, 31]. Norway is about to establish regional screening programs, based on recommendations from the Norwegian Directorate of Health.

In the Oslo region, our cohort found similar presence and distribution of the DR grades in the minority women compared to the general population of this region as recently screened in another study [32]. The population of Oslo having a high percentage of people of non-European background seems to be more vulnerable to develop DR and, therefore, needs a tighter control of their DM and DR. It is known that ethnic minority groups have a higher prevalence of DM than the rest of the population in Norway [16, 18, 19]. Clinical experience shows that DM patients with ethnic minority background, in particular, have higher risk for not undertaking regular eye examinations and progression to severe DR. Furthermore, the estimated number of undiagnosed DM is of high public health importance, as years of delayed diagnosis equal years of DR and other complications to develop.

Regular screening is, therefore, crucial to prevent vision loss from DR (especially in higher risk groups) [33]. However, traditional screening methods, with dilated eye examination by ophthalmologist, using either ophthalmoscopy or stereoscopic 7 field images, face challenges, in addition to limited access to skilled personnel and time-consuming exams. Leveraging AI in ophthalmology presents a promising solution to the growing need for accurate and efficient screening.

In a large study, conducted through the English Diabetic Eye Screening Programme, the AI system (RetCAD v.2.1.0, Netherlands) was tested on 9817 patients. The AI was found to have 69.7% sensitivity and 92.2% specificity for detecting any diabetic retinopathy. It performed even better in mild cases, with 95.4% sensitivity and 92.0% specificity [15]. In a recent review paper, Rajesh et al. [34] analysed prospectively fully automated and autonomous algorithms using non-dilated 45° fundus images. All 6 of them had sensitivity and specificity > 85% (IDx-DR (renamed LumineticsCore), EyeArt, AYE-DS (FDA approved), SELENA, Google, VoxelCloud Retina and AIDRScreening system). Yet, compared to humans, they were found to be more sensitive to poor image quality and more often gave ungradable results. Since they were tested on different sets of images using different grading scales, and performed on a population with different retinal pigmentations [35] they were difficult to compare to each other [34]. Tufail et al. compared 3 of the AI-based grading systems in the same population in England: 20,258 patients were examined, and 102,856 images analysed as part of the U.K. National Health Service Diabetic Eye Screening Programme to clarify whether three ARIAS systems (automated retinal image analysis systems) iGradingM, Retmarker and EyeArt could help to replace human graders. Both Retmarker and EyeArt appeared to have high enough sensitivity for referable DR (EyeArt 93.8% (92.9-94.6%), Retmarker 85.0% (83.6-86.2%)) and also to be cost-effective choice comparing to human graders (gold standard) [14, 36]. Similar to our findings, the English Diabetic Eye Screening Programme conducted on 30,000 patients, found the sensitivity of referable DR to be 95.7% (CI 94.8 to 96.5) and the specificity of referable DR 54.0% (CI53.4 to 54.5) compared to human graders. The study concluded that AI is a safe way to screen for high-risk retinopathy in real-world screening [37]. On the other hand, in a large study conducted on 5 anonymized algorithms, Lee et al. [38] showed varying performance among these, with most not surpassing human graders. Interestingly, different models performed differently in cohorts with various demographics and dilation procedures. This study emphasizes the need for further research to guide clinicians in choosing effective AI algorithms for their practice [38].

Existing literature on whether AI screening is cost-effective is inconsistent, and factors like location and how it is implemented seem to have a significant impact [34]. In countries with high income (HIC), cost of human graders is higher, so it is easier for AI algorithm to price lower [38, 39]. Studies from Thailand and China, considered to be low- and middle-income countries (LMIC), found AI to be more cost-effective [40, 41]. However, other studies from China [42] and Brazil [43] have claimed the opposite, arguing that factors like “years without blindness” and better compliance with patient referrals could contribute to AI being more cost-effective than humans [42]. The latest study from Singapore suggested that a combination AI/ human, where AI analysed images first and humans graded the positive cases, appeared to be most cost-effective [44].

Assessing the cost-effectiveness of using AI for DR screening compared to manual screening by ophthalmologists deserves critical discussion in Norway and wider. Systematic evaluation of screening programs aimed at mitigating the impact of diabetes should be assessed for their impact on health outcomes, cost-effectiveness, and health equity. This is crucial for evidence-based healthcare decision-making [14, 34].

The role of AI in DR screening in case of proven specificity, and sensitivity holds great potential in healthcare as a valuable addition, given the growing interest in AI applications in medicine. In our pilot study, a high inter-rater agreement between manual/ophthalmologist and AI-based EyeArt grading of DR could be achieved, which is in line with previous studies on similar or larger cohorts [45].

The cost-minimization evaluation comparing the costs of manual grading by ophthalmologists and AI-driven grading gives healthcare policymakers the basis for the potential cost-saving benefits of AI screening in Norway or other countries where DRG reimbursement codes for AI-based screening does not exist.

Many immigrants of non-European origin have a higher susceptibility to develop T2D. This applies to Norway as well, with its prominent immigration from the Indian subcontinent, Asia and Africa. This highlights the importance of tailored healthcare strategies for different population groups and optimized DR screening programs.

A primary limitation of our study is the small sample size, posing an increased risk of insufficient study power to detect a true, clinically relevant effect (Type II error) or falsely detecting a non-existent effect (Type I error). Additionally, the limited generalizability of findings to the broader Norwegian population is noteworthy, as the study population of minority women may not be representative of the overall demographic, potentially introducing selection bias. Moreover, the non-random selection of the study participants restricts our ability to analyze causal relationships, despite this not being the primary focus of the study. The use of a small sample compromises internal and external validity, with external factors, such as participant characteristics and environmental influences, potentially impacting study results but not comprehensively controlled. Other limitation of our study include the use of only one type of non-mydriatic fundus camera, which may limit the generalizability of the results. The use of single-field color fundus photographs might also lead to a certain rate of missed-diagnoses. In light of these limitations, it is crucial to interpret the results cautiously.

Further research is needed to investigate if different camera models would yield different outcomes. Additionally, larger sample sizes and diverse ethnic populations should be included to enhance the study’s findings and overcome selection bias.

## Conclusion

Our study provides evidence that AI could effectively screen/detect referable DR in real-world setting in a highly sensitive and specific manner. Such system can have higher accuracy when deployed at the primary-level than in tertiary-level healthcare settings.

 The study also highlights the potential of AI in addressing these challenges, backed by empirical data and economic analysis. Further well-designed, multicenter studies with larger sample size with diverse ethnic backgrounds populations should be included to enhance the study’s findings and overcome selection bias and to make informed decisions on healthcare strategies in DR screening.

## Data Availability

The datasets during and/or analyzed during the current study are available from the corresponding author on reasonable request.
